# Attention Based Visual Analysis for Fast Grasp Planning With a Multi-Fingered Robotic Hand

**DOI:** 10.3389/fnbot.2019.00060

**Published:** 2019-07-31

**Authors:** Zhen Deng, Ge Gao, Simone Frintrop, Fuchun Sun, Changshui Zhang, Jianwei Zhang

**Affiliations:** ^1^Department of Informatics, University of Hamburg, Hamburg, Germany; ^2^Department of Computer Science and Technology, Tsinghua University, Beijing, China

**Keywords:** grasp planning, grasp type, visual attention, deep learning, multi-fingered robotic hand

## Abstract

We present an attention based visual analysis framework to compute grasp-relevant information which helps to guide grasp planning using a multi-fingered robotic hand. Our approach uses a computational visual attention model to locate regions of interest in a scene and employ a deep convolutional neural network to detect grasp type and grasp attention point for a sub-region of the object in a region of interest. We demonstrate the proposed framework with object grasping tasks, in which the information generated from the proposed framework is used as prior information to guide grasp planning. The effectiveness of the proposed approach is evaluated in both simulation experiments and real-world experiments. Experimental results show that the proposed framework can not only speed up grasp planning with more stable configurations, but also handle unknown objects. Furthermore, our framework can handle cluttered scenarios. A new Grasp Type Dataset (GTD) which includes six commonly used grasp types and covers 12 household objects is also presented.

## 1. Introduction

Imagine a toddler is in front of a table top with several objects, very likely he or she would interact with those objects by trying to pick up the red mug either by the handle or the rim, or trying to grasp the green ball. The ability to rapidly extract relevant information from visual input is an important mechanism and natural behavior for humans to conduct various activities. The majority of visual analysis approaches for grasp planning with multi-fingered robotic hands follow a pipeline containing object localization, recognition and representation (Schwarz et al., 2017). For most existing approaches, finding a target object in a scene is the first step for robotic grasping. However, reliable object detectors, such as deep-learning based approaches require vast amounts of training data, as well as good hardware to achieve a reasonable time performance for robotic applications, while handcrafted feature based approaches can not handle the dynamics in real life scenarios.

This paper proposes an attention based visual analysis framework which directly locates sub-regions of objects as regions of interest (ROIs), and generates grasp-relevant information from visual data inside the ROIs for grasp planning with a multi-fingered robotic hand. The proposed learning framework is inspired by psychological studies which demonstrated that humans combine early bottom-up processing with later top-down processing to visually analyze the scene (Theeuwes, 2010; Awh et al., 2012). The bottom-up process starts with sensor input data and is completely stimulus-driven, while the top-down process extracts relevant information, which may be influenced by prior experience and semantics. In particular, a computational attention model is used to process visual data and outputs a pixel-precise saliency map, from which salient regions are selected for further processing. Inside those salient regions, the grasp type and grasp attention point are predicted by a network. The grasp attention point indicates the location on the object surface where the robot plans the grasp. Finally, this information is used to guide grasp planning with a multi-fingered robotic hand.

Grasp type and grasp attention point convey useful information for planning the configuration of a robotic hand. In the computer vision community, most previous works sample human hand pose with a motion tracking system and use it to detect hand grasp types (Rogez et al., 2015; Cai et al., 2017). In the robotics community, there are few previous approaches that try to integrate grasp type detection into robotic grasp planning (Harada et al., 2008; Vahrenkamp et al., 2018). In those works, only two kinds of grasp types, i.e., power and precision (Napier, 1956), are considered, which is not sufficient for exploring the potential of multi-fingered robot hands. Moreover, the desired grasp type is determined manually for robotic hands. In terms of visual analysis, there are approaches which use visual analysis to define heuristics or constraints for grasp planning (Hsiao et al., 2010; Aleotti and Caselli, 2012; Vahrenkamp et al., 2018). In comparison to those approaches, there are three main differences: (1) our approach learns features directly from raw sensor data, while most of the previous approaches use handcrafted features; (2) six grasp types are considered while the previous approaches only consider two grasp types. (3) Most of the previous works only focus on visual analysis by using computer vision techniques. This work uses the results of the visual analysis for grasp planning with multi-fingered robotic hands. The effectiveness of the proposed framework is evaluated in a real-world object grasping experiment.

In this paper, we address the problem of visual analysis of natural scenes for grasping by multi-fingered robotic hands. The objective is to compute grasp-relevant information from visual data, which is used to guide grasp planning. A visual analysis framework which combines a computational visual attention model and a grasp type detection model is proposed. A new Grasp Type Dataset (GTD) which considers six commonly used grasp types and contains 12 household objects is also presented.

The rest of the paper is organized as follows: section 2 presents related work. Section 3 introduces the architecture and main components of the proposed visual analysis framework. Grasp planning is described in section 4. Experimental results are presented in section 5. Finally, the conclusion and future work are discussed in section 6.

## 2. Related Work

Stable grasping is still a challenge for the robotic hands, espectically multi-fingered robotic hand, since it usually require to solve a complex non-conex optimization problem (Roa and Suárez, 2015; Zhang et al., 2018). Information extracted from visual analysis can be used to define heuristics or constraints for grasp planning. Previous grasp planning methods can be divided into geometric-based grasping and similarity-based grasping. In geometric-based grasping (Hsiao et al., 2010; Laga et al., 2013; Vahrenkamp et al., 2018), geometric information of the object is obtained from color or depth images, and it is used to define a set of heuristics to guide grasp planning. Hsiao et al. (2010) proposed a heuristic which maps partial shape information of objects to grasp configuration. The direct mapping from object geometric to candidate grasps is also used in Harada et al. (2008) and Vahrenkamp et al. (2018). Aleotti and Caselli (2012) proposed a 3D shape segmentation algorithm which firstly oversegments the target object, and candidate grasps are chosen based on the shape of the resulted segments (Laga et al., 2013). In similarity-based approaches (Dang and Allen, 2014; Herzog et al., 2014; Kopicki et al., 2016), the similarity measure is calculated between the target object and the corresponding object model from human demonstrations or simulation. The candidate grasp is then queried from datasets based on similarity measures. Herzog et al. (2014) defined an object shape template as the similarity measure. This template encodes heightmaps of the object observed from various viewpoints. The object properties can also be presented with semantic affordance maps (Dang and Allen, 2014) or probability models (Kroemer and Peters, 2014; Kopicki et al., 2016). Geometric-based approaches usually require a multiple-stage pipeline to gather handcrafted features through visual data analysis. Due to sensor noise, the performance of the geometric-based grasping is often unstable. Meanwhile, similarity-based methods are limited to known objects and can not handle unknown objects. In contrast to previous methods, our method increases grasp stability by extracting more reliable features from visual data using deep networks, meanwhile, it is able to handle unknown objects.

Many saliency approaches have been proposed in the last two decades. Traditional models are usually based on the feature integration theory (FIT) (Treisman and Gelade, 1980) to compute several handcrafted features which were fused to a saliency map, e.g., the iNVT (Itti et al., 1998; Walther and Koch, 2006) and the VOCUS system (Frintrop, 2006). Frintrop et al. (2015) proposed a simple and efficient system which computes multi-scale feature maps using Difference-of-Gaussian (DoG) filters for center-surround contrast and produces a pixel-precise saliency map. Deep learning based saliency detection mostly relies on high-level pre-trained features for object detection tasks. Those learning-based approaches require massive amounts of training data (Huang et al., 2015; Li et al., 2016; Liu and Han, 2016). Kümmerer et al. (2015) used an AlexNet (Krizhevsky et al., 2012) pretrained on Imagenet (Deng et al., 2009) for object recognition tasks. The resulting high-dimensional features are used for fixation prediction and saliency map generation. Since most of the deep-learning based approaches have a central photographer bias which is not desired in robotic applications, we choose to use a handcrafted feature based approach which gathers local visual attributes by combing low-level visual features (Frintrop et al., 2015).

## 3. Attention Based Visual Analysis

**Figure 1:**
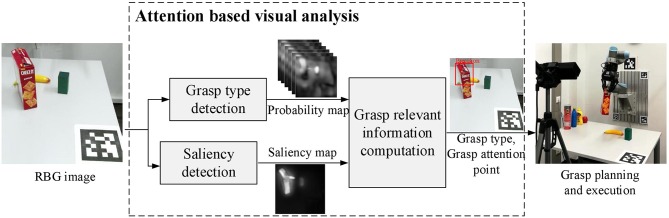
The proposed attention based visual analysis framework. With an input RGB image, a ROI is selected using the saliency map produced by a Saliency detection model. Inside the ROI, grasp type and grasp attention point are computed based on the six probability maps produced by the Grasp type detection network. The obtained information containing grasp type and grasp attention point is then used as a prior to guiding grasp planning. The planned grasp is executed by a robotic hand to verify its quality.

The proposed framework contains two main components, a computational visual attention model which gathers low-level visual features and selects ROIs for further processing, and a grasp type detection model which learns higher level features and produces grasp-relevant information in the ROIs. Figure 1 illustrates an overview of the proposed attention based visual analysis framework.

### 3.1. Computational Visual Attention Model

The pixel-level saliency map is computed using the computational visual saliency method VOCUS2 (Frintrop et al., 2015). In principle, any saliency system which has a real-time capability and does not have a center-bias could be used. Center bias gives preference to the center of an image, which is not desired in robotics applications. Unfortunately, this excludes most deep-learning based approaches since they are usually trained on large datasets of Internet images, which mostly have a central photographer bias. Therefore, the VOCUS2 system was chosen, which belongs to the traditional saliency systems with good performance on several benchmarks. In VOCUS2, an RGB input image is converted into an opponent-color space including intensity, red-green and blue-yellow color channels. DoG contrasts are computed with twin pyramids, which consist of two Gaussian pyramids—one for the center and one for the surround of a region—which are subtracted to obtain the DoG contrast. Finally, the contrast maps are fused across multiple scales using the arithmetic means to produce the saliency map.

**Figure 2:**
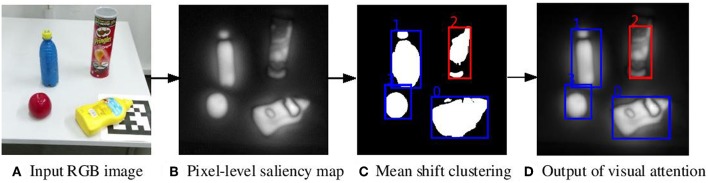
Saliency region detection with the visual attention model. **(A)** The input RGB image, **(B)** the pixel-level saliency map, **(C)** the result after clustering, **(D)** the output. The red rectangle denotes the selected ROI which has the highest average saliency value. The blue rectangles denote the candidate ROIs for objects. The numbers are indices for bounding boxes.

Given the produced saliency map, the pixels of the saliency map are clustered using Mean Shift (Comaniciu and Meer, 2002) to form saliency regions. The salient region with the highest average salient value is selected as the ROI, and it is passed to the next stage for further processing. Figure 2 shows an example of the saliency region detection. The visual attention model takes the RGB image shown in Figure 2A as input and produces the saliency map shown in Figure 2B. After clustering, the desired saliency region is determined, as shown in Figure 2C.

### 3.2. Grasp Type Detection

**Figure 3:**
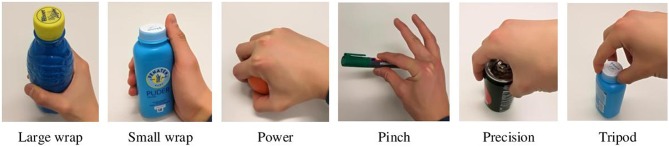
The proposed six commonly used grasp types.

Grasp type is a way of representing how a hand handles objects. Typically, the robotic grasps are divided into power and precision grasp (Napier, 1956). Power grasp uses the fingers and palm to hold the object firmly, while precision grasp only uses fingertips to stabilize the object. However, this two-categories grasp taxonomy is not sufficient to convey information about hand configuration. Feix et al. (2016) introduced a GRASP taxonomy in which 33 different grasp types used by humans are presented. All the 33 different grasp types are classified into four groups: prismatic power, circular power, intermediate, prismatic precision, circular precision. Considering the kinematic limitations of the robotic hand as well as Feix's GRASP taxonomy, we extend the above two-categories grasp taxonomy into six commonly used grasp types: large wrap, small wrap, power, pinch, precision, and tripod. Figure 3 illustrates the proposed grasp taxonomy.

In order to detect grasp types directly from visual data, we refer to the architecture proposed by Chen et al. (2018). This architecture is based on a deep convolutional neural network [VGG-16 (Simonyan and Zisserman, 2015)] and uses atrous convolution for signal down sampling. Since an object may support multiple feasible grasp types (Feix et al., 2016), the grasp type detection is a multi-label detection. Hence, we modify the output layer of the network and do not use the additional fully connected Conditional Random Field (CRF). Corresponding to the six grasp types, the modified network predicts six pixel-level probability maps with the same resolution as the input image. In order to train the modified network for grasp type detection, this paper introduces a grasp type detection (GTD) dataset, in which 12 household objects are used and all the instances are annotated following the proposed six grasp types. The details of the GTD dataset are provided in section 5.1. This work uses a cross-entropy function to define the loss function which is defined as

(1) $$\begin{array}{l} L\left(\theta \right)=\overset{h}{\underset{i=1}{\sum }}\overset{w}{\underset{j=1}{\sum }}\underset{s\in S}{\sum }logP\left(y_{i,j}^{s}|I,\theta \right) \end{array}$$

where $y_{i,j}^{s}\in \left\{0,1\right\}$ indicates if the pixel *y*_*i, j*_ belongs to the grasp type *s* ∈ *S* or not. *S* = [1, 2, ⋯ , 6] is the index of the six grasp types. *I* denotes an RGB image with height *h* and width *w*. θ is the weight of the proposed detection model.In this work, the cross-entropy based on the sigmoid function is defined in Equation (2), where *f* is the trained network.

(2) $$\begin{array}{l} P\left(y_{i,j}^{s}|I;\theta \right)=1/\left(1+exp\left(-f\left(y_{i,j}^{s}|I;\theta \right)\right)\right) \end{array}$$

Given an RGB image *I* with height and width *h* × *w* as input, our network outputs pixel-level probability maps *P*(*Y*|*I*) for each grasp type *s* ∈ *S*, where $Y={\left\{y_{i,j}^{s}\right\}}_{i=1:h,j=1:w}$. The predicted probability of pixel {[*i, j*]_*i* = 1:*h, j* = 1:*w*_} belonging to the grasp type *s* is denoted by $y_{i,j}^{s}$. With the pixel-level probability maps, the probability *P*(*Y*^*s*^|*O*) is computed by summing the predicted probabilities of all the pixels inside the ROI *O* (defined in section 3.1), as shown in Equation (3). The grasp type with the highest probability is used as the final grasp type s^*^.

(3) $$\begin{array}{l} P\left(Y^{s}|O\right)=\frac{1}{h_{O}\times w_{O}}\overset{h_{O}}{\underset{i=1}{\sum }}\overset{w_{O}}{\underset{j=1}{\sum }}P\left(y_{i,j}^{s}|x_{i,j}\right),\forall s\in S. \end{array}$$

**Figure 4:**
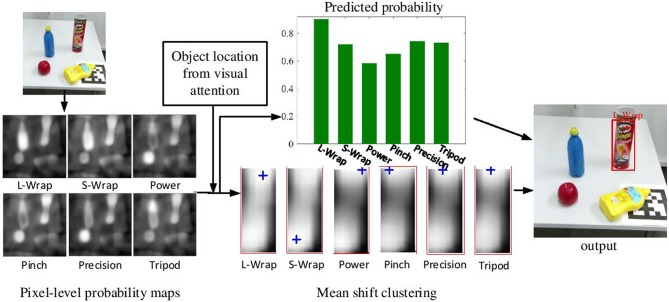
The detection process of grasp type and grasp attention point. Six pixel-level probability maps corresponding to the six grasp types are first computed from the grasp type detection network. Given the object location computed by the visual attention model, these probability maps are clustered. Then the predicted probability of each grasp type and the location of its grasp attention point are computed. Finally, the grasp type with the highest probability and its grasp attention points are determined.

After determining the best grasp type s^*^, we need to localize the grasp attention point for the grasp type s^*^ inside *O*. In order to find a stable grasp attention point *p*, subregions with higher predicted probabilities are clustered. Mean Shift (Comaniciu and Meer, 2002) is used to find a grasp attention point *p* in *O*. Multiple clusters with multiple centers are produced, and the cluster center with the highest probability is selected as the grasp attention point *p*. Finally, the grasp relevant information $\Omega =\left\{O,s^{*},p_{o}\right\}$, i.e., ROI *O*, the grasp type s^*^ and the grasp attention point *p*_*o*_, are generated from the proposed visual analysis framework. Figure 4 illustrates the detection process of grasp type and grasp attention point.

## 4. Grasp Planning With Grasp-Relevant Information

The objective of grasp planning is to find the feasible grasp configuration for a stable grasping. Hence, grasp planning in this work is formulated as an optimization problem. A search based algorithm exploiting grasp-relevant information Ω generated from the proposed visual analysis framework is proposed to find the grasp configuration with high grasp quality. In this work, the search of the feasible grasp configuration is processed from two steps: (1) the formation of the initial grasp configuration based on the grasp-relevant information, (2) the determination of the feasible grasp configuration by the local transformation.

In the first step, we take advantage of the grasp-relevant information $\Omega =\left\{o,s^{*},p_{o}\right\}$ to determine the initial grasp configuration and the number of the required finger. The initial grasp configuration of the robotic hand is defined as follows: (1) The number of needed fingers is selected according to the grasp type s^*^ and the gripper; (2) The grasp center *p*_*h*_ is set to be a point that deviate a initial offset *d*_*init*_ from the 3D grasp attention point $p_{o}^{\prime }$ which is obtained from 2D grasp attention point *p*_0_ using frame transformation; (3) The hand palm is controlled to approach the grasp attention point. Using a multi-fingered robotic hand to grasp objects typically requires the relative pose between the object and the robotic hand, as well as the hand joint configuration. Due to the high dimensionality of the robotic hand and partially observability of objects, it is challenging to find the optimal contact points on the object surface to form a grasp configuration. In this work, we exploit the concept of Opposition introduced by De Souza et al. (2015) to execute the grasp configuration. The robotic hand is controlled to reach the target pose and close the two finger groups to grasp an object.

Next, A local search method is used to find the grasp configuration with the highest quality in a grasp search space. Due to the existence of uncertainties, the defined pre-grasp configuration may fail to grasp objects. Hence, a local search is used to find the grasp configuration with higher quality. During searching, the pre-grasp configuration is used as the initial grasp configuration. We sample a set of candidate grasps with coordinate transformation. The search space is a 4 dimensional space, *S* = {*d*, α, β, γ}, where *d* = *d*_*init*_ ± Δ*d* is the offset of the 3D grasp attention point $p_{o}^{\prime }$. Δ*d* is a pre-defined searching range. {α, β, γ} denote the searching ranges of the rotate angles in the *X*, *Y* and *Z* axes of the hand coordinate, respectively. During the search process, all the candidates are evaluated by using force-closure method (Suárez et al., 2006). The force-closure method has been widely used in grasp planning, which measures the grasp quality through the evaluation of certain geometric relations of the contact points. A grasp is force-closure if a hand can exert arbitrary force on the grasped object through a set of the contact point. After the grasp quality measure, the grasp configuration with the highest quality is chosen for object grasping. Finally, during executing candidate grasps, the fingers move to contact with the object surface and hold it. The robotic arm lifts the object to finish the grasping task.

Algorithm 1 shows the process of the grasp planning procedure.

**Algorithm 1:** Attention based visual analysis for grasp planning

1: Requires: a computational saliency model, a grasp type detection model
2: Acquire an RGB image *I* of the table scene.
3: Visual analysis framework returns the grasp-relevant information $\Omega =\left\{O,s^{*},p_{o}\right\}$.
4: Using the information Ω to initialize the pre-grasp configuration of the hand.
5: Using a local search method to find a list of feasible candidate grasps.
6: Find the grasp configuration with the highest quality from all the feasible grasps
7: Execute the grasp operation by using robotic hands.

## 5. Experimental Results

### 5.1. Dataset and Implementation

**Figure 5:**
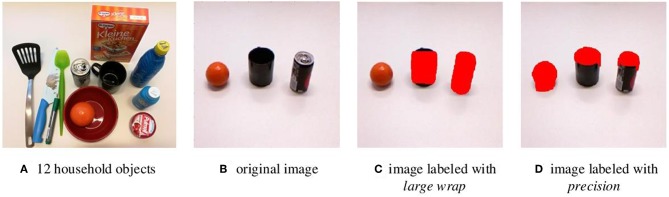
Illustration of GTD dataset. **(A)** Twelve household objects contained in the GTD. **(B)** The original image. **(C)** A labeled image with large wrap. **(D)** A labeled image with precision. Pixels that belong to a grasp type are marked with color and others are background.

Existing datasets, such as the Yale human grasping datasets (Bullock et al., 2015) and the UT grasp dataset (Cai et al., 2015), are used for the analysis of human hand behavior. These datasets are not suitable for the grasp planning with robotic hands. Hence, we introduce a new grasp type detection (GTD) dataset specified for robot grasping. The GTD dataset contains RGB-D^1^ images and ground-truth grasp type labels. There are 11,000 annotated images with resolution 640 × 480. In this dataset, six commonly used grasp types were considered and 12 household objects with various shape attributes were chosen, as shown in Figure 5A. A MATLAB GUI is designed to manually annotate grasp types on collected data. According to the GRASP taxonomy defined in Feix et al. (2016), object parts in images were labeled with different grasp types which enable multi-label detection, as shown in Figures 5B,C. The GTD dataset was split randomly into a training set (90%) and a testing set (10%). The training parameters of the grasp type detection model are set as follows: the initial learning rate was 0.00001, and a step delay policy is used to lower the learning rate as the training progresses.Stochastic gradient descent (SGD) method with a momentum rate of 0.9 is used.

### 5.2. Evaluation of Grasp Type Detection

We first evaluated the accuracy of the grasp type detection on the proposed GTD dataset. For comparison, another network based on the Segnet architecture introduced in Badrinarayanan et al. (2017) is trained and evaluated. Segnet has an encoder-decoder architecture and is widely used for image segmentation. For pixel-level multi-label detection, we modified the output layer of the Segnet network as introduced in subsection 3.2. The same training and testing procedures are used for both networks described in section 5.1. Table 1 shows the Intersection-over-union (IoU) of the two networks. Our approach achieves a higher average detection accuracy and outperforms the segnet-based network by 10%.

**Table 1:** Performance on GTD dataset (IoU).

	**L-wrap**	**S-wrap**	**Power**	**Pinch**	**Precision**	**Tripod**	**Average**
Ours	**0.63**	**0.58**	**0.71**	0.56	**0.61**	**0.52**	**0.60**
Segnet-based	0.51	0.56	0.41	**0.61**	0.46	0.48	0.50

*Bold numbers denote better accuracies*.

**Figure 6:**
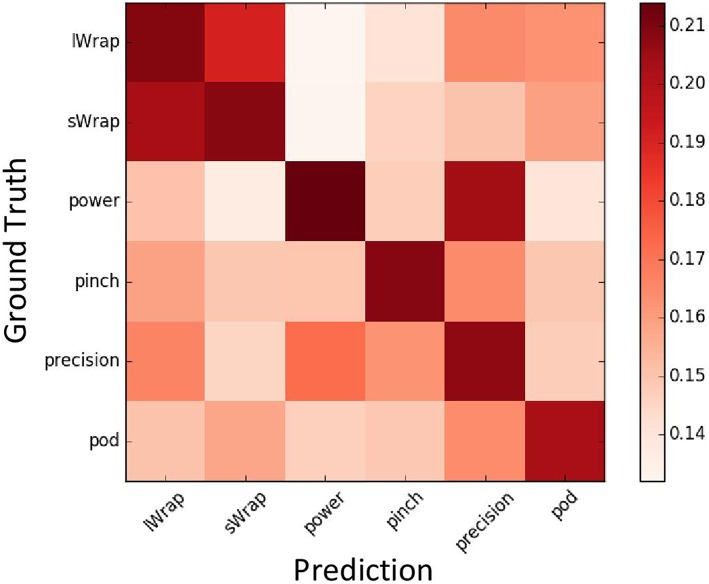
The confusion matrix of the six grasp types.

A confusion matrix (Figure 6) is used to evaluate the overall quality of detected the grasp type. Since the network predicts six labels corresponding to six grasp types for each pixel, each row of the matrix shows the predicted probabilities of each grasp type for one ground truth label. It shows that the proposed method is able to predict correct grasp types with the highest probability since the diagonal elements have the highest values. It is worth mentioning that several off-diagonal elements also have rather high values. For example, the prediction results for Power type also show a high probability for Precision, which means those two grasp types are easily mislabeled by the proposed method. The reason is that those two types have a high correlation and share many similar characters. Hence, the confusion matrix can also help to discover the similarity among grasp types.

### 5.3. Grasp Planning in Simulator

**Figure 7:**
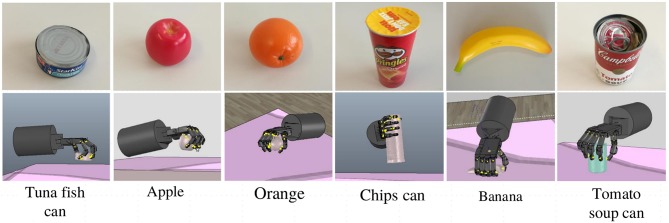
Examples of object grasping by the Shadow Dexterous Hand in the simulator.

The proposed visual analysis framework was further evaluated in object grasping tasks. We implemented a grasping simulation based on the V-REP^2^, which is a physical simulator that supports rapid verification, to conduct this experiment. The grasping experiments were performed on a Shadow Dexterous Hand^3^, a five-fingered robotic hand which is an approximation of a human hand. During simulations, the hand configuration and the contact force between the Shadow Dexterous Hand and objects were simulated in real-time, which were used for measuring the qualities of candidate grasps. In order to evaluate the performance of the visual analysis framework for grasp planning, we compared the proposed planning method with the method proposed by Veres et al. (2017). Veres et al. used a method which randomly samples a set of candidate grasps based on the normal of the object surface and then ranked all the candidates to find the best one. Since there is no grasp type provided in this method, we use the commonly used power type for the Shadow Dexterous Hand to grasp objects. In this comparison experiment, six objects were selected, as shown in Figure 7. Ten trials are tested for each object. For each trial, an object is placed on the table top and a depth sensor is used to capture the RGB-D image of the table scene. Then, the grasp configuration of the Shadow Dexterous Hand is planned in the simulator. The maximum number of search attempts for both methods is limited to 40. For each object, the success rate of object grasping and the average number of search attempts needed for finding a feasible grasp are shown in Table 2.

**Table 2:** Performance of the proposed grasp planning.

	**Ours**		**Veres et al. (****2017****)**	
**Object**	**Success rate**	**Search attempt**	**Success rate**	**Search attempt**
Tomato soup can	8/10	2.5	8/10	20
Tuna fish can	9/10	8.7	5/10	23.6
Banana	9/10	2.1	5/10	21.6
Apple	9/10	2.5	8/10	27.5
Orange	8/10	2.8	7/10	19.4
Chips can	10/10	2.7	10/10	11.4
Average	88.3%	3.5	71.6%	20.5

It can be seen that the proposed method obtained a higher success rate of grasping than the random search method. Moreover, the number of search attempts by the proposed planning method is only 17.0% of the search attempts by the random search method. It shows that the grasp-relevant information generated helps to reduce the search time needed for grasp planning and to more accurately find the feasible grasp configuration in the search space. It is worth mentioning that the random search method with a power type easily fails at grasping some small objects, such as the banana and the tuna fish can. This limitation does not occur in the proposed planning method since a feasible grasp type is predicted before grasping. Hence, for multi-finger robotic hands, objects with different shape attributes should be handled with different grasp types.

We also noticed that there are several failures of object grasps using the proposed planning method. The main reason for the failures is because the predicted grasp attention point on the object surface is too close to the table top. Since the environmental constraints are not considered in this work, the Shadow Dexterous Hand will collide with the table and fail to grasp the object. In the future, it will be beneficial also to consider the environment and task constraints.

**Figure 8:**
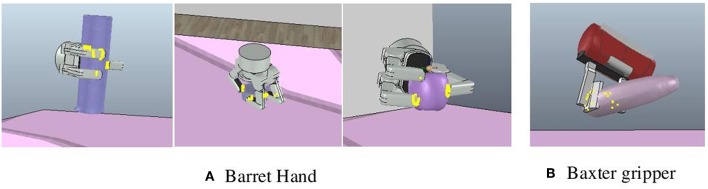
Examples of object grasping. **(A)** Objects grasped by the Barrett hand. **(B)** An object grasped by the Baxter gripper.

In order to further evaluate the generalization of the proposed framework, we also tested our framework with a 3-fingered Barrett hand^4^ and a 2-fingered Baxter gripper^5^, Figure 8 shows some results of object grasping. In this experiment, the 2-fingered Baxter gripper only used the pinch type to grasp objects. On average, Barrett hand has 90% success rate with four search attempts while Baxter gripper has 100% success rate with 1.4 search attempts.

To further verify the effectiveness of the grasp planning with prior information, we compared with the work from Ciocarlie and Allen (2009). This work searches a grasp configuration for dexterous robotic hands in a hand posture subspace which is determined by using grasp synergies. In their work, the grasp planner only results in a power type, which means their grasp planner may fail to grasp small objects. Another limitation of their grasp planner is that it needs a long search time for finding a feasible solution, with over 70,000 attempts for each plan, and an average running time of 158 s (Ciocarlie and Allen, 2009). Compared with their work, our method requires fewer search attempts and enables the robotic hand to grasp objects with different grasp types.

### 5.4. Real-World Robotic Experiment

**Figure 9:**
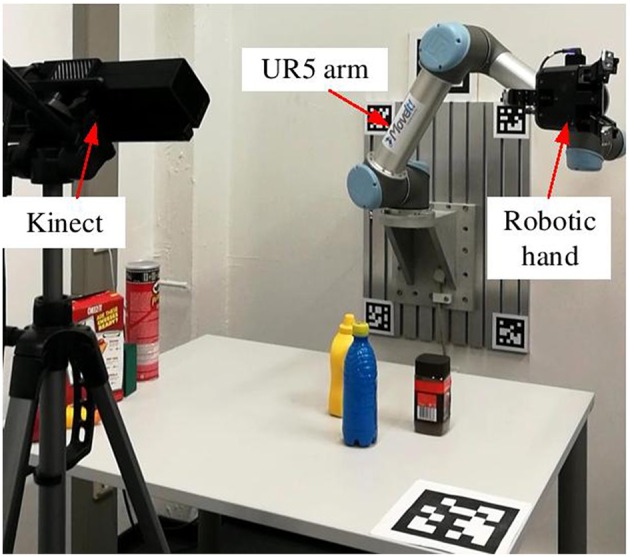
Experimental setup with a UR5 arm and a three-fingered robotic hand.

**Figure 10:**
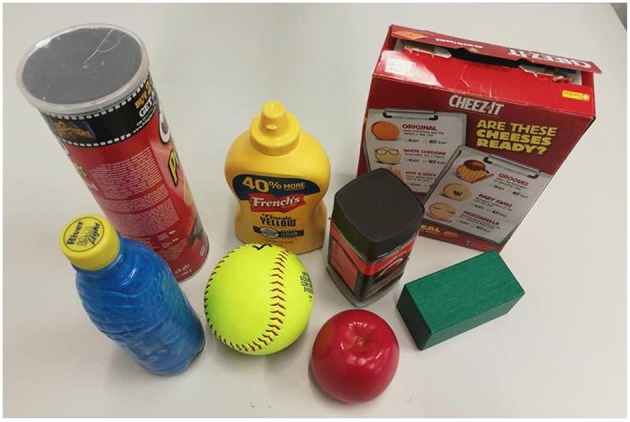
Eight different objects for robotic experiments.

The robotic experiments were conducted using the six DOF UR5 robot^6^ and the three-fingered Robotiq gripper^7^. Figure 9 shows the experimental setup for the object grasping tasks. A Kinect sensor was used to capture the RGB-D image of the table scenes. Eight objects selected from YCB object set (Calli et al., 2015) were used for the evaluation, as shown in Figure 10. It contains six unknown objects comparing to our dataset (Figure 5). In the object grasping experiments, we adopted the following procedure. Multiple objects were randomly selected and placed on the table. The proposed visual analysis framework took the image captured by Kinect as input and outputted the grasp-relevant information. Then, the grasp configuration was planned by taking advantage of this computed information and sent to the UR5 robot for grasping. A video is provided as Supplementary Material.

**Figure 11:**
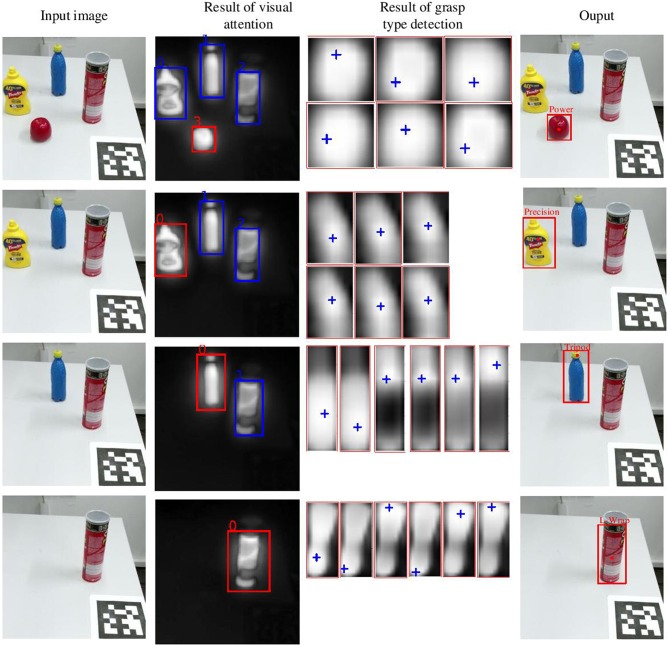
Example of the visual analysis on various objects. The first column is the input RGB image. The second column is the pixel-level saliency map, in which the red rectangle denotes the selected ROI. The third column is six pixel-level probability maps which describe the results of grasp type detection. The six probability maps from top left to bottom right corresponds to the six grasp types (i.e., large wrap, small wrap, power, pinch, precision, and tripod). The cross in the probability maps denote the cluster centers which is considered as the grasp attention point. The last column is the output of the visual analysis.

Figure 11 shows the process of attention based visual analysis. Given an input RGB image, the ROI denoted by a rectangle in the saliency map is firstly selected by the attention model. Meanwhile, six pixel-level probability maps are obtained from the grasp type detection model. The grasp attention point denoted by the cross in each probability map is obtained by clustering. Finally, the grasp type with the highest probability in the ROI is selected. As it is shown in Figure 11, our system is also able to produce grasp type and grasp attention point results on unknown objects.

**Figure 12:**
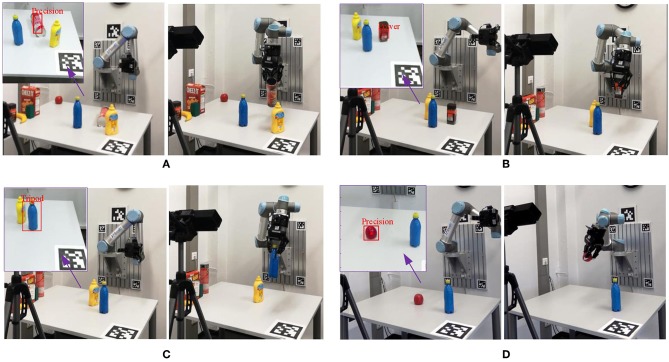
Examples of object grasping using the UR5 robot. In each subfigure, the left showed the analyzed results and the right showed the robot grasped the object. **(A)** Grasping of a chip can With Precision grasp type, **(B)** grasping of a coffee bottle with power grasp type, **(C)** grasping of a bottle with Tripod grasp type, **(D)** grasping of an apple with precision grasp type.

The performance of the whole system is evaluated based on object grasping tasks. Four trails were tested for each object and a total of 32 trails were implemented. Because the robotic gripper only had three finger, we consider large wrap and small wrap equivalent, and consider precision and tripod equivalent. So the numbers of the used finger for precision and tripod were same. The experimental results were that 28 successful graspings out of 32 trails (87.5%). Basically, the proposed method enabled the robotic hand to find the feasible grasp configuration and successfully grasp it. Figure 12 shows some examples of the object grasping using the proposed framework. As we can see, the grasp-relevant information generated from the proposed framework was used as prior information to guide the grasp formation. For each frame, ROI localization takes 1.8 s, grasp type detection takes 6.5 s and the complete process takes 8.5 s on average. The proposed framework is implemented in python and runs on a 2.50 GHz Intel i5 CPU.

It is worth mentioning that several failures of object grasping have occurred. As in simulation experiments, when grasping the small object (e.g., an apple), the planned grasp pose was too close to the table, the UR5 robot failed to find a feasible kinematic solution. Another cause was that the proposed visual attention method sometimes only locate a small region of an object and a feasible grasp configuration cannot be found. This is caused by low color contrast between the object and its background. It also occurred that the object fell out of the gripper during lifting. It was caused by the uncertainty from the object weight. In the future, it will also be beneficial to incorporate grasp adaptation into the proposed framework.

## 6. Conclusion

This paper proposes an attention based visual analysis framework, which computes grasp-relevant information directly from visual data for multi-fingered robotic grasping. By using the visual framework, an ROI is firstly localized by a computational attention model. The grasp type and grasp attention point on object segment presented in the ROI is then computed using a grasp type detection model, which is used as prior information to guide grasp planning. We demonstrated that the proposed method is able to give a good prediction of grasp type and grasp attention point. Furthermore, the performance of the proposed visual analysis framework has been evaluated in object grasping tasks. Compared to previous methods without prior, the information generated from the visual analysis can significantly speed up grasp planning. Moreover, by using a feasible grasp type, the success rate of the grasping is also improved. Results show that the proposed framework helps the robotic systems to know how and where to grasp objects according to attributes of sub-regions of objects. Since our method does not rely on object detection, it can also handle unknown objects.

For future work, several aspects will be considered: first, the current framework is goal-driven, and it only learns how to grasp an object, so it will be interesting to extend the proposed framework into a task-driven framework, e.g., grasping in human-robot handover task. Second, currently the choice of grasp type and grasp attention point only depends on the attributes of sub-regions of objects. Since grasp planning is also affected by environment and task constraints, those constraints will be taken into consideration.

## Data Availability

The datasets for this study can be found in the GTD dataset^8^.

## Author Contributions

ZD and GG designed the visual analysis framework. SF provided the visual attention model for saliency detection. ZD and GG designed the experiments and carried out the experiments. ZD analyzed the experimental results. ZD and GG wrote the manuscript. FS, CZ, and JZ assisted the manuscript writing.

### Conflict of Interest Statement

The authors declare that the research was conducted in the absence of any commercial or financial relationships that could be construed as a potential conflict of interest.

## Footnotes

^1^We use only RGB data in this paper, and plan to exploit the depth data in the future.

^2^http://www.coppeliarobotics.com/

^3^https://www.shadowrobot.com/products/dexterous-hand/

^4^https://www.barrett.com/about-barrethand/

^5^https://www.rethinkrobotics.com/baxter/

^6^https://www.universal-robots.com/products/ur5-robot/

^7^https://robotiq.com/products/3-finger-adaptive-robot-gripper

^8^https://tams.informatik.uni-hamburg.de/people/deng/index.php?content=research

## Supplementary Material

Video S1 Attention based visual analysis for grasp planning.
